# In depth annotation of the *Anopheles gambiae* mosquito midgut transcriptome

**DOI:** 10.1186/1471-2164-15-636

**Published:** 2014-07-29

**Authors:** Alejandro Padrón, Alvaro Molina-Cruz, Mariam Quinones, José MC Ribeiro, Urvashi Ramphul, Janneth Rodrigues, Kui Shen, Ashley Haile, José Luis Ramirez, Carolina Barillas-Mury

**Affiliations:** Laboratory of Malaria and Vector Research, National Institute of Allergy and Infectious Diseases, National Institutes of Health, Rockville, MD USA; Bioinformatics and Computational Biosciences Branch, National Institute of Allergy and Infectious Diseases, National Institutes of Health, Rockville, MD USA

## Abstract

**Background:**

Genome sequencing of *Anopheles gambiae* was completed more than ten years ago and has accelerated research on malaria transmission. However, annotation needs to be refined and verified experimentally, as most predicted transcripts have been identified by comparative analysis with genomes from other species. The mosquito midgut—the first organ to interact with *Plasmodium* parasites—mounts effective antiplasmodial responses that limit parasite survival and disease transmission. High-throughput Illumina sequencing of the midgut transcriptome was used to identify new genes and transcripts, contributing to the refinement of *An. gambiae* genome annotation.

**Results:**

We sequenced ~223 million reads from *An. gambiae* midgut cDNA libraries generated from susceptible (G3) and refractory (L35) mosquito strains. Mosquitoes were infected with either *Plasmodium berghei* or *Plasmodium falciparum*, and midguts were collected after the first or second *Plasmodium* infection. In total, 22,889 unique midgut transcript models were generated from both *An. gambiae* strain sequences combined, and 76% are potentially novel. Of these novel transcripts, 49.5% aligned with annotated genes and appear to be isoforms or pre-mRNAs of reference transcripts, while 50.5% mapped to regions between annotated genes and represent novel intergenic transcripts (NITs). Predicted models were validated for midgut expression using qRT-PCR and microarray analysis, and novel isoforms were confirmed by sequencing predicted intron-exon boundaries. Coding potential analysis revealed that 43% of total midgut transcripts appear to be long non-coding RNA (lncRNA), and functional annotation of NITs showed that 68% had no homology to current databases from other species. Reads were also analyzed using *de novo* assembly and predicted transcripts compared with genome mapping-based models. Finally, variant analysis of G3 and L35 midgut transcripts detected 160,742 variants with respect to the *An. gambiae* PEST genome, and 74% were new variants. Intergenic transcripts had a higher frequency of variation compared with non-intergenic transcripts.

**Conclusion:**

This in-depth Illumina sequencing and assembly of the *An. gambiae* midgut transcriptome doubled the number of known transcripts and tripled the number of variants known in this mosquito species. It also revealed existence of a large number of lncRNA and opens new possibilities for investigating the biological function of many newly discovered transcripts.

**Electronic supplementary material:**

The online version of this article (doi:10.1186/1471-2164-15-636) contains supplementary material, which is available to authorized users.

## Background

The *Anopheles gambiae* mosquito is the primary vector of malaria in sub-Saharan Africa, where this disease causes 139.2 million infections and 542,360 deaths per year [1]. Malaria control has relied mainly on vector control—with insecticides and insecticide-impregnated nets—and on antimalarial therapy of infected humans. These strategies have reduced malaria prevalence and transmission, but development of insecticide resistance in the vector and of drug resistance in the parasite limit their effectiveness [2]. The *Plasmodium* parasite population undergoes a major bottleneck in the mosquito, making it an attractive target for novel strategies to disrupt disease transmission. Mosquitoes become infected when they ingest host blood containing *Plasmodium* gametocytes, and fertilization takes place giving rise to a motile ookinete that invades the mosquito midgut epithelia. Usually only a few ookinetes (<5) are able to complete their development and multiply in the mosquito. The mosquito midgut is the first epithelial barrier that parasites must traverse to complete their development, and cellular responses of invaded midgut cells have been shown to limit parasite survival [3].

Sequencing of the *An. gambiae* genome was a landmark that provided a powerful platform to advance our understanding of the biology of this mosquito vector and its role in malaria transmission. The genome, published in 2002, was done using shotgun sequencing; gene prediction and annotation was done, in large part, *in silico* based on homology with known genes from other species [4]. This is a powerful approach, but it has some limitations, as there can be errors in the predicted gene models and many transcripts—for example, those unique to *An. gambiae*—could be missed. In *Drosophila*, whole-genome tiling-array expression analysis revealed that the initial genome sequence annotation had missed 30% of the transcripts [5], and in the *P. falciparum* malaria parasite, the first genome sequence contained errors in 25% of the predicted gene models [6].

Here we report the in-depth transcriptome analysis of the *An. gambiae* mosquito midgut using RNA-seq by Illumina sequencing with the goal of discovering new transcripts and improving the genome annotation, especially of midgut-expressed genes, as interaction of *Plasmodium* with this organ is critical for the parasite to establish an infection. RNA-seq has been used successfully to study transcriptional differences of previously annotated genes, between chemosensory appendages and whole body [7] and between insecticide resistant and susceptible *An. gambiae* [8]. We explored the mosquito midgut transcriptome of two *An. gambiae* strains*,* the L3-5 refractory strain that was selected to melanize *Plasmodium,* and the *An. gambiae* G3 susceptible strain under different physiological conditions. Mosquitoes were infected with different *Plasmodium* species with a variable level of compatibility [9] to identify the maximum number of transcripts induced in response to infection. Samples were collected 24 h after feeding, a time when epithelial cells are responding to ookinete invasion. L3-5 females were infected with gametocytes from two different *P. falciparum* lines: 7G8 from Brazil, which is melanized and is almost completely eliminated, and the 3D7 strain that survives very well in this strain [10]. *An. gambiae* G3 was infected with *Plasmodium berghei*, and midguts were collected 24 h after the first infection (naïve response) or after a second infection (primed response) [11]. We report a high-throughput RNA-seq analysis using a genome-based sequence assembly that generated novel transcript models and doubled the number of known transcripts for *An. gambiae*. Furthermore, several of the predicted transcript models were experimentally validated*.* A transcriptome using a *de novo* assembly—a strategy that can be particularly useful in organisms whose genome has not been sequenced—was also performed and compared with the genome-based approach. Finally, variant analysis of the sequence reads identified many new polymorphisms that could be useful for future genetic studies in this disease vector.

## Results

### Genome-based analysis of illumina reads

**Figure 1:**
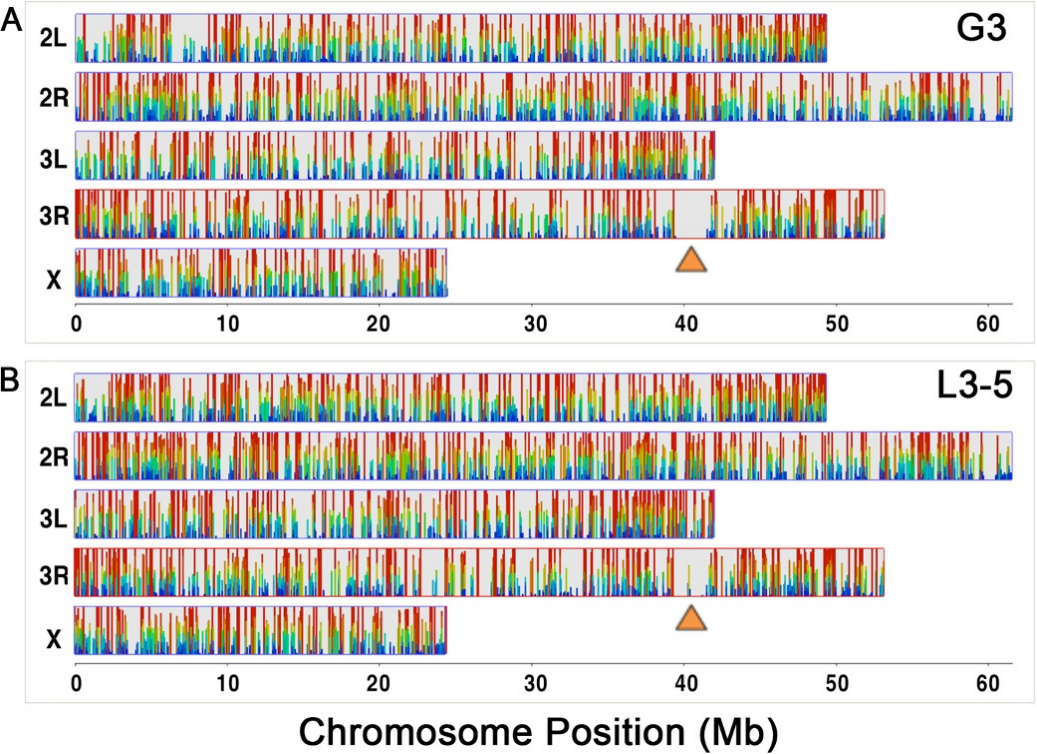
**Heat map of coverage of the Illumina reads for the** ***Anopheles gambiae*** **midgut transcriptome.** **A)** Coverage of Illumina reads obtained for *An. gambiae* G3 strain along the *An. gambiae* genome. **B)** Coverage of Illumina reads obtained for *An. gambiae* L3-5 strain along the *An. gambiae* genome. Within a data zoom of 3, the colors scale linearly from blue to green to red (low to high coverage). The gap region of poorly expressed genes in chromosome 3R corresponds to the heterochromatic region (orange arrow) near subdivision 35B/C.

Illumina sequencing generated a total of ~223 M reads from midguts of *A. gambiae* G3 and L3-5 strains. Raw reads were processed for quality using Btrim [12] to generate ~51-bp high-quality reads (Additional file 1: Figure S1). A total of 119.4 M high-quality reads for the G3 and 103.5 M from the L3-5 strain were mapped to the reference *An. gambiae* genome using TopHat [13]. Most of the reads from G3 (91.4%) and L3-5 (90.4%) mapped to the *An. gambiae* genome. The sequence reads obtained from both strains were distributed across the three *An. gambiae* chromosomes (Figure 1), indicating no sequencing bias according to genome location. The gap region of poorly expressed genes in chromosome 3R (Figure 1) corresponds to the heterochromatic region (orange arrow) near subdivision 35B/C [14]. Most of the mapped reads (89%) mapped to exons of previously annotated genes, covering at least half of the exon sequences.

Transcript models for *An. gambiae* G3 and L3-5 strains were generated with Cufflinks [15] using the mapped reads. The normalized read coverage values, fragment per kilobase of exon per million fragments mapped (FPKM), for all transcripts obtained were analyzed by frequency, showing that a large number of transcript models had no coverage (Additional file 1: Figure S2). This is expected, as the program also considers all the known transcript models based on the annotated genome, and not all of them are transcribed in the adult female midgut. To discard transcript models that had no read coverage (FPKM = 0) or low coverage, a threshold was set and only transcripts with FPKM ≥ 1 were considered for the rest of the analysis (see transcript experimental validation below). G3 and L3-5 transcript models had similar median read coverage values of 4.4 and 3.7 FPKM, respectively (Additional file 1: Figure S3). Cufflinks generated a total of 22,889 unique midgut transcripts (with FPKM ≥ 1) from both strains combined (Additional file 2: Table S1). These transcripts were compared to the reference genome (AgamP3.6) [16] with Cuffcompare (Table 1; Additional file 2: Table S2). A total of 5,483 transcripts (23.9%) had a complete match to previously annotated transcripts, while 17,406 (76%) were potentially novel. Of these novel transcripts, 8,623 (49.5%) aligned with annotated genes either in exons (5,942), in introns of the same (2,550) or opposite strand (131) (directionality was predicted based on the consensus sequence of the splice junctions), or they appeared to be isoforms (4940) or pre-mRNAs (470) of reference transcripts. Of the potentially novel transcripts 8,783 (50.5%) map to regions of the genome between annotated genes, and we will refer to them as novel intergenic transcripts (NITs).

**Table 1:** **Distribution of** ***Anopheles gambiae*** **midgut transcripts by Cufflinks class code**

Transcript class codes	Number	Percentage
**GENIC**	5483	23.95
Complete match	5483	23.95
Novel isoform	4940	21.58
Within reference intron	2550	11.14
Read mapping errors	15	0.07
Overlap	517	2.26
Pre-mRNA	470	2.05
Exonic overlap to opposite strand	131	0.57
**Subtotal**	**14106**	**61.62**
**INTERGENIC**		
Polymerase run-on	1821	7.96
Unknown Intergenic	5450	23.81
Repeat	707	3.09
Multiple classifications	805	3.52
**Subtotal**	**8783**	**38.38**
**TOTAL**	**22889**	**100**

Number of *Anopheles gambiae* midgut transcripts for each Cufflinks class code and as a percentage of the total.

NITs can be subdivided into four different Cufflinks transcript codes: unknown intergenic transcripts (5450), transcripts that are within 2 Kb of a reference transcript and could be polymerase run-ons (1821), intergenic transcripts with repeats (707), and intergenic transcripts with multiple classifications (805) (Table 1). Interestingly, 82% of NITs had an open reading frame that can code for a peptide of 50 amino acids or more. Independent evidence was obtained for the existence of 3,514 (40%) NITs, because they either had high sequence homology by BLAST (e ≤ 10^−10^) to the *An. gambiae* expressed sequence tag database (AgEST) (3,005 NITs) or to a Diptera protein database (1,072 NITs), and some transcripts had matches to both databases (563 NITs) (Additional file 2: Table S2). The other 5,269 NITs (60%) had no match in any database and appear to be unique to *An. gambiae* (Additional file 2: Table S2).

### Independent validation of transcript models

In total, 56 transcript models were tested by reverse transcription polymerase chain reaction (RT-PCR) in independent *An. gambiae* midgut samples to confirm their presence in the midgut transcriptome (Additional file 3: Table S3). Transcript models for validation were chosen among low FPKM ranges, 0.86–111.56, where 18 transcripts corresponded to previously known genes in the reference genome and 38 corresponded to transcripts that had not been previously described. Of the 56 transcripts tested, 47 (84%) were confirmed by RT-PCR (Additional file 3: Table S3). The graphic representation of the region in the genome where the sequence reads of three novel intergenic transcripts (NITs) (TCONS_00022174, TCONS_00032244 and TCONS_00022201) that were experimentally validated map is shown in Additional file 1: Figure S4. A total of 31 potential novel splice junctions in annotated genes was also experimentally tested (Additional file 4: Table S4), and most of them (84%) were confirmed using PCR and Sanger sequencing. A graphic respresentation of the genomic location of three new exons in a predicted transcript (TCONS_00023667) for the *Anopheles gambiae* cyclin A gene (AGAP012413) that was experimentally validated is shown in Additional file 1: Figure S5. The high rate of independent confirmation of expression or novel splice junctions indicates that most of the predicted novel transcripts are real. Finally, validation of a larger number of NITs was also obtained by microarray analysis. An independent sample of *An. gambiae* G3 strain midgut RNA—collected 26 h after feeding on uninfected human blood—was hybridized with a microarray that included 2,050 probes for NITs, and a positive hybridization signal could be detected for 800 (39%) of them (Additional file 5: Table S5).

### Functional annotation of transcripts

**Figure 2:**
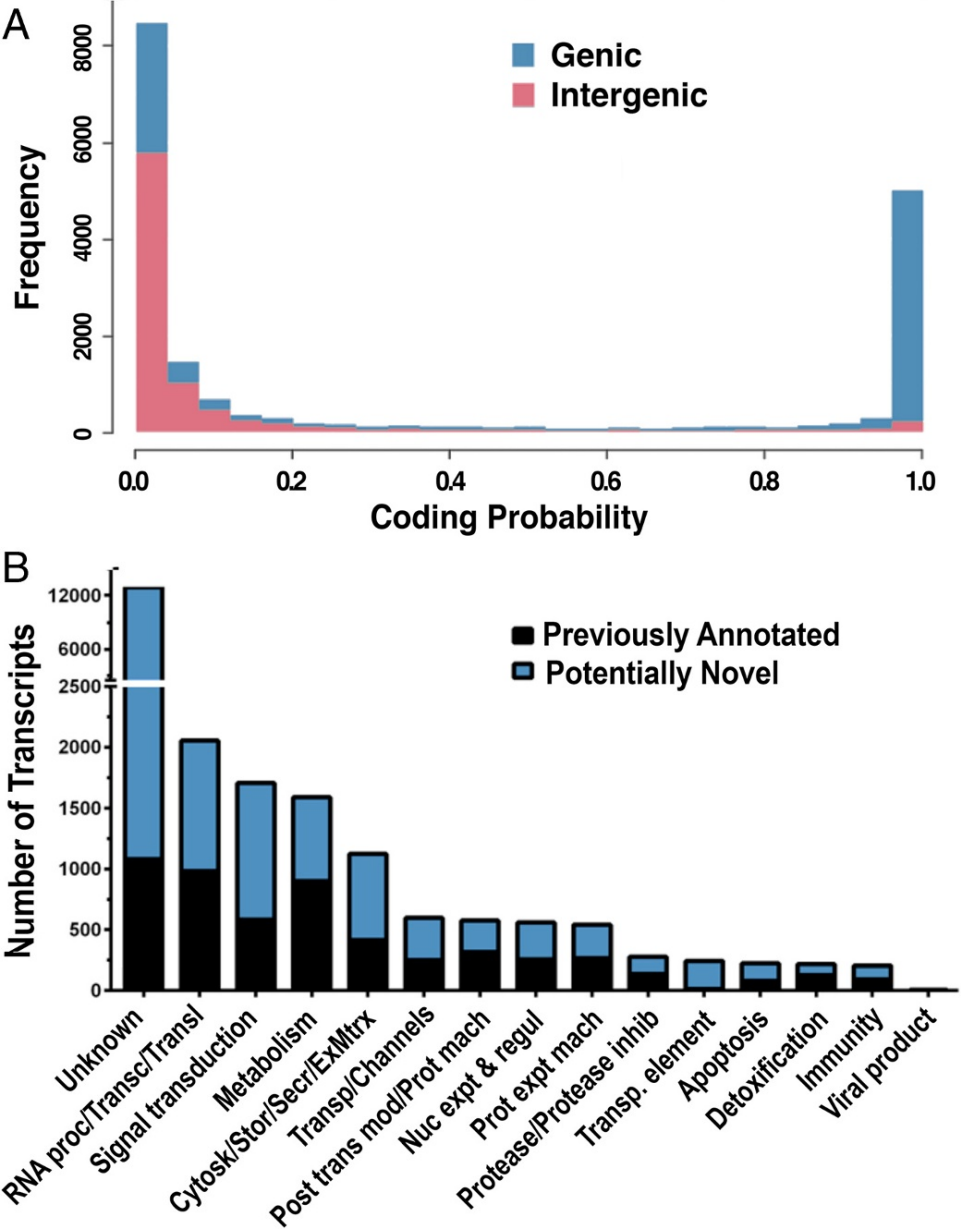
**Coding probability of** ***Anopheles gambiae*** **midgut transcripts and functional classification of** ***An. gambiae*** **midgut transcripts generated by a genome-based analysis. A)** Intergenic transcripts (light pink color) and genic (blue) show a bimodal distribution that defines two major populations of transcripts with different coding probability. **B)** Number of potentially novel and previously annotated *An. gambiae* transcripts were functionally classified by BLAST to different databases. Abbreviated titles are “RNA Processing/Transc/Transl”: RNA Processing, Transcription, Translation; “Cytosk/Stor/Secr/ExMtrx”: Cytoskeletal, Secretion, Extracellular Matrix; “Transp/Channels”: Transporters and Channels; “Post trnsl mod/Prot mach”: Post-translational modification and proteasome machinery; “Nuc export & Reg” Nuclear Export and Regulation. “Protease/Protease inhib”: Protease and protease inhibitors; “Transp. Element”: Transposable Element.

The coding potential of the midgut transcripts was analyzed with CPAT taking into account open reading frame (ORF) size, ORF coverage, hexamer usage bias, and the Fickett TESTCODE statistic [17]. Overall, 43% of the total midgut transcripts identified appeared to be long non-coding RNA (lncRNA) (Additional file 2: Table S2). LncRNA were less frequent in the annotated gene transcripts—3,008 of 14,106 (21.3%) (Figure 2A; Table 2), where most of them (1,616) were located within reference introns (Table 2). The proportion of lncRNAs is much higher, 6,855 of 8,783 (77.2%), in NITs and includes the following Cufflinks classes: unknown genes (4,335), potential polymerase run-on (1,511), transcripts with repeats (377), and multiple classifications (632) (Table 2).

**Table 2:** **Distribution of** ***Anopheles gambiae*** **midgut lncRNA by Cufflinks class code**

Transcript class codes	Amount	LncRNA	Percentage
**GENIC**			
Complete match	5483	321	5.8
Novel isoform	4940	601	1.2
Within reference intron	2550	1616	63.3
Read mapping errors	15	10	66.7
Overlap	517	151	29.2
Pre-mRNA	470	219	46.6
Exonic overlap to opposite strand	131	90	68.7
**Subtotal**	**14106**	**3008**	**21.3**
**INTERGENIC**			
Polymerase run-on	1821	1511	83.0
Unknown intergenic	5450	4335	79.5
Repeat	707	377	53.3
Multiple classifications	805	632	78.5
**Subtotal**	**8783**	**6855**	**78.05**
**TOTAL**	**22889**	**9863**	**43.1**

Number of *An. gambiae* midgut long non-coding RNA (lncRNA) by Cufflinks class codes and as a percentage of the class code transcript total.

The *An. gambiae* midgut transcript models obtained (22,889) (Additional file 2: Table S1) were functionally annotated by multiple BLAST analysis against 12 databases (see Methods, Additional file 2: Table S2). We were able to annotate 9,908 (43.3%) of them within a designated functional class (e ≤ 10E^−10^) (Figure 2B, Additional file 6: Table S6). Of the transcripts without functional annotation, 13% were conserved (e ≤ 10E^−10^) across several insect genus such as *Drosophila*, *Aedes*, and *Culex,* but the rest appear to be exclusive to anophelines. We found that 20% of transcripts with a complete match to known reference transcripts (5,483) had no identifiable functional annotation, while within the potentially novel transcripts (17,406), the unknown function class increased to 68.4%. The four most abundant functional classes were, in order of abundance, RNA processing/transcription/translation; signal transduction, metabolism, and cytoskeletal/storage/secretion/extracellular matrix/adhesion (Figure 2B, Additional file 6: Table S6). All functional classes presented potentially novel transcripts—in some cases in higher proportion than those previously annotated (Figure 2B, Additional file 6: Table S6). The immunity class consisted of 204 transcripts; 112 of them were novel, and 92 had been previously annotated. Of the novel transcripts, 88 (96%) were new isoforms of previously annotated transcripts, 8 were potential pre-mRNA transcripts, 6 had exonic overlaps to reference transcripts, 5 were exonic or intron overlaps to the opposite strand (directionality was predicted based on the consensus sequence of the splice junction), and 3 were intergenic.

### Analysis of Illumina reads using *de novo*assembly

A reference genome sequence is not available for many relevant insect vectors, and high-throughput transcriptome analysis can be very useful to begin to characterize candidate genes and develop new tools, such as microarrays, that would make it possible to assess broad transcriptional responses to specific physiologic conditions or experimental treatments. We carried out a *de novo* assembly of our reads, independent of the *An. gambiae* genome sequence, and compared the output of this strategy with the genome-based analysis using TopHat/Cufflinks as described. This alternative *de novo* assembly was also used as a complementary approach to identify new reads and maximize the discovery of novel transcripts.

The RNA-seq reads for G3 and L3-5 were compiled together and put through an assembly by short sequences (ABySS) [18, 19] pipeline (see Methods). The *de novo* strategy assembled 67,011 contigs of which 49,969 (75%) aligned to the *An. gambiae* genome (BLAST cut off e-value ≤ 1 × E^−20^) (Additional file 7: Tables S7 and S8). Of the 17,042 *de novo* contigs that did not align to the genome, 83% had matches to an Apicomplexa protein database and are likely to be either *P. berghei* or *P. falciparum* transcripts, while 17% were neither *An. gambiae* nor Apicomplexan sequences and probably represent transcripts from bacteria, mouse, or human cells from the blood meal.

**Figure 3:**
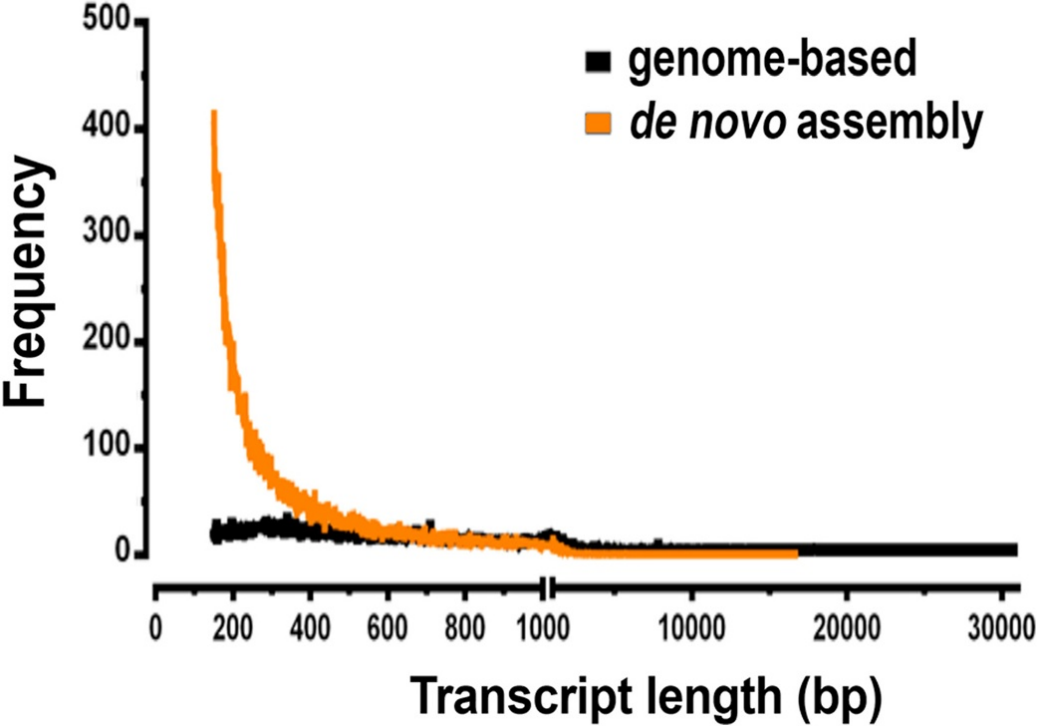
**Frequency of** ***Anopheles gambiae*** **midgut transcripts by length.** Transcripts were generated either by a genome-based strategy using TopHat/Cufflinks (black line) or by a *de novo* strategy using ABySS (orange line).

Overall, the *de novo* strategy generated more contigs, but they were shorter (about 3 fold) than the genome-based assembled transcripts (Figure 3; Table 3). This strategy was able to detect 98% of the transcripts from annotated reference genes detected by the genome-based strategy and detected 1009 additional transcripts from reference genes. When we compared the transcripts predicted from both methods, 59% of *de novo* transcripts aligned with transcripts obtained with the genome-based strategy, while 74% of the transcripts obtained with the genome-based analysis aligned with transcripts from the *de novo* assembly (BLAST cut off e-value ≤ 1 × E^−20^) (Table 3). It is important to keep in mind that for the genome-based analysis only transcripts with FPKM > 1 were included in this comparison, and we know that up to 78% of transcripts with low read coverage (FPKM < 1) could be validated by qRT-PCR. In other words, several transcripts with low expression were probably eliminated when we established this quality threshold for the transcripts predicted using the genome-based methodology.

**Table 3:** **Alignment comparison of** ***de novo*** **and genome-based assembly strategies for the** ***Anopheles gambiae*** **midgut transcriptome**

			Reference genes detected	
Query	# Transcripts	AVG Length (BP)	Unique	Shared
***De novo***	67011	678	1009	6881
**Genome Based**	20273	2039	167	

Alignment comparison was done using BLAST with *de novo* contigs as the query and with genome-based assembly transcripts as the subject and vice versa.

### Variant analysis

**Figure 4:**
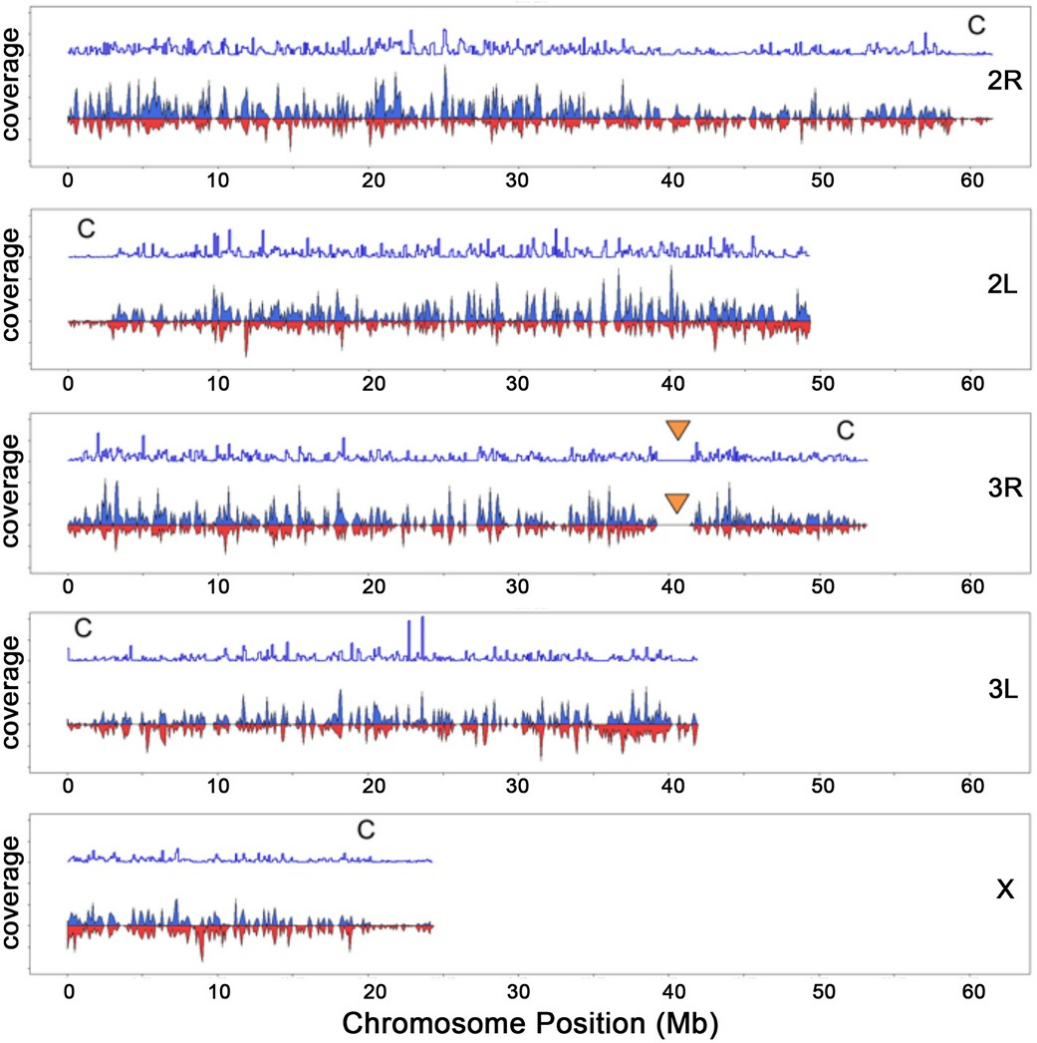
**Density of variants and expressed genes across 100-kb loci for the midgut transcripts for each** ***Anopheles gambiae*** **chromosomal arm.** Variant density (blue filled graph) and gene density (red filled graph) shows variants across the entire genome with a tendency to decrease toward centromeric regions (“C” label). The gap region of no variants or expressed genes in Chromosome 3R corresponds to the heterochromatic region (orange arrow) near subdivision 35B/C. A normalized ratio of variants to gene density (blue line graph) shows regions of high polymorphism and regions of low polymorphism.

The G3 and L3-5 *An. gambiae* midgut transcript reads were preprocessed using Picard, and sequence polymorphisms or variants were discovered and annotated using the genome analysis toolkit (GATK) and a program for annotating and predicting the effects of single nucleotide polymorphisms (snpEFF) (see methods). Overall, 160,742 variants were detected with respect to the genome (Agam3.6) of the pink-eyed laboratory strain (PEST) of *An. gambiae*. Of these 119,344 were not reported in the dbSNP database (version 125) and are therefore potential new variants. The *An. gambiae* G3 and L3-5 strains presented 123,517 and 81,825 variants with respect to the reference genome, respectively (Figure 4; Additional file 7: Table S9). The *An. gambiae* G3 and L3-5 strains shared 44,600 of the variants. The G3 strain has 1 variant every 2,209 bp while the L3-5 strain has 1 variant every 3,335 bp. The L3-5 strain was selected from the G3 *An. gambiae* strain for refractoriness to *Plasmodium cynomolgi* [20] and is therefore expected to have less diversity than the parental G3 strain.

**Figure 5:**
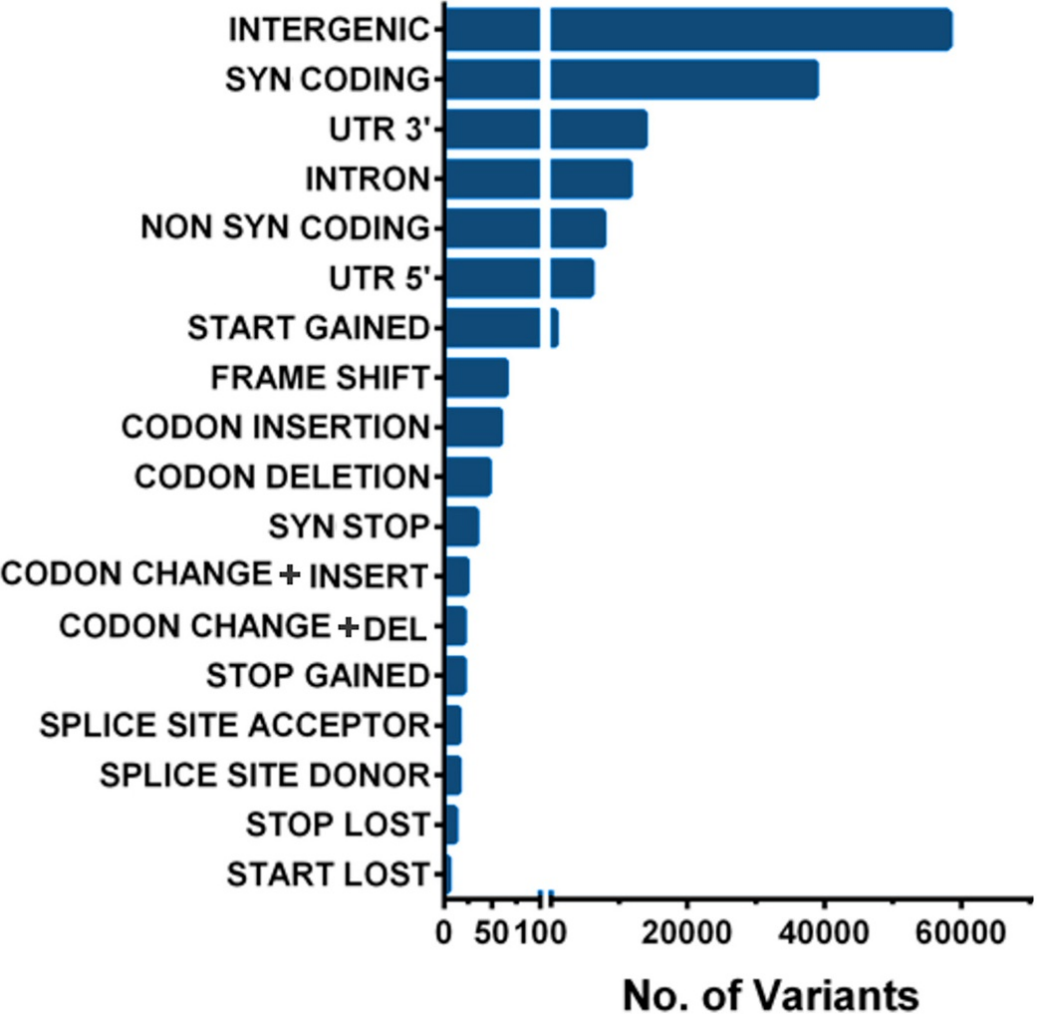
**Annotation of the detected** ***Anopheles gambiae*** **variants in mapped reads from the midgut transcriptome.** The type of variant in either the G3 or L3-5 *An. gambiae* strain vs. the pink-eyed laboratory strain of *An. gambiae* (PEST; AgamP3.6) reference genome. Variant annotation was performed using a program for annotating and predicting the effects of single-nucleotide polymorphisms (snpEFF).

Variants found in the transcriptome were distributed along the three *An. gambiae* chromosomes, with higher frequency toward telomeres and lower frequency toward centromeres (Figure 4). Annotation of the *An. gambiae* transcriptome variants showed that 57,988 (42.3%) are located in intergenic regions of the annotated reference genome (Figure 5, Additional file 7: Table S9), and many of them are probably present in non-coding RNAs that tolerate more variation than coding RNAs. Intergenic transcripts have a higher level of variation (12.5 variants/transcript) than transcripts from annotated genes (5.3 variants/transcripts). SNPs were frequently found in synonymous coding sequences (38,700 = 28.4%), 3’ untranslated regions (19,626 = 14.4%), and intronic regions (11,501 = 8.4%) (Additional file 8: Table S10) that are predicted not to affect the amino acid sequence of the translated products. We also identified 8,646 (6.3%) SNPs predicted to change the encoded proteins. They either introduce a new start or stop codon, insert or delete a codon, a frame shift—by generating a novel donor or acceptor splice site, or result in loss of a start or stop codon (Additional file 8: Table S10). Non-synonymous variants were found in most functional gene classes including genes involved in general metabolism, cytoskeletal structure, and extracellular matrix formation (Additional file 8: Table S10).

## Discussion

We report the assembled high-throughput transcriptome of the *An. gambiae* midgut. A total of 22,889 unique transcripts expressed in the *An. gambiae* midgut were detected. The number of potential novel transcripts identified (17,406) is surprising, as it doubles the number of transcripts currently reported in the whole genome database (14,974). Of these potential novel transcripts, 49.5% align partially to reference genome transcripts and represent new isoforms of 3,819 known genes; while the other 50.5% map to intergenic regions of the annotated genome (novel intergenic transcripts [NITs]) and define 7,745 novel genes. We present several lines of evidence that confirm the existence of a good portion of the NITs. First, 40% of them have highly homologous sequences in either the *An. gambiae* EST database or the Diptera database, indicating that similar transcripts have been previously found. Second, RT-PCR testing confirmed expression of 84% of transcripts, and sequencing confirmed 84% of novel splice junction. Previous reports have shown that microarrays are less sensitive and often unable to detect low abundant transcripts [21], and some predicted transcripts may only be expressed in midguts from *Plasmodium*-infected mosquitoes. In spite of these limitations, we were able to confirm expression of ~800 NITs, 39% of a subset of 2,050 novel transcripts tested in a microarray analysis of midgut mRNA expression in females fed uninfected blood. Together, these data provide strong evidence that most of the novel transcripts reported are real and of high quality.

Interestingly, the protein coding probability analysis indicated that a large portion of the transcripts detected (43%) appear to be lncRNA (Figure 2A). In fact, most of the NITs (78%) appear to be lncRNA, compared with 21% of the transcripts from annotated genes (genic); however, 82% of the NITs contain ORFs at least 50 amino acids long, suggesting that some transcripts could code for short peptides. Functional annotation of the NITs done by BLAST revealed that 68.4% of them do not have homology to any functional database. This percentage is significantly higher than the 20% of unknown function in the previously annotated transcripts. The larger percentage of unknown function within the NITs suggests that some of these transcripts may be unique to anophelines*.* Our finding of a large number of new transcripts and noncoding RNA in *An. gambiae* is consistent with some of the main findings of the ENCODE project [22]. This project identified 73,325 novel transcripts in intergenic or antisense regions of the reference human genome, based on sequences from human cell lines, and increased the number of annotated transcripts by 45%. Moreover, 6,205 polyadenylated lncRNAs were found in genic regions of the human genome [22]. It appears that a large portion of the eukaryotic genome is transcribed, and while previous efforts have focused on short non-coding RNA, the function of most of the newly discovered lncRNA is still unclear [23, 24]. Recent reports indicate that lncRNAs are involved in diverse biologic functions, such as chromosome X inactivation in females [25, 26] and regulation of inflammatory responses [27]. Our detection of a large number of new transcripts is also consistent with a previous RNA-seq analysis of transcriptional differences between chemosensory organs and whole body *An. gambiae*, in which only 57.4% of the reads mapped to annotated genes in the AgamP3.6 genome [7].

The *de novo* strategy assembled 67,011 unique contigs, and 49,969 (75%) of them mapped to the *An. gambiae* genome. The number of transcripts found in the *de novo* strategy is in the same order of magnitude of transcripts reported (95,747) for a sugar-fed male and female *An. gambiae* transcriptome by RNA-seq assembled also with a *de novo* strategy [28]. The genome-based strategy used here produced less fragmented models that were on average three times longer than those from the *de novo* strategy (Figure 3). The genome-based analysis carried out with TopHat/Cufflinks can build transcript-spanning non-overlapping reads if there is a reference transcript; this generates larger and less fragmented transcripts [15]. Because the *de novo* strategy does not require a reference genome for contig assembly, it would still generate contigs in regions where genomic scaffolds may be missing in poorly sequenced or partially assembled genomes. The *de novo* assembly also has the advantage that it does not require a reference genome and is able to find most of the transcripts generated by the genome-based analysis. There were 371 transposable element (TE) transcripts in the RNAseq *de novo* assembly that were identified using a RPS-Blast search against a compiled database of mosquito TE's from pfam and Repbase (*e* value of 1e-15 or lower, Additional file 7: Table S8). In particular, long stretches (>1000 bp) were found for several Jockey, Copia and Outcast elements. Transcripts coding for near full-length transposases of Class II elements of the mariner, gambol and PIF elements were also found. Most of them appear to be functional genes, as they did not contain stop codons that are often found in pseudogenes. The presence of these transcripts could indicate active transposition of elements in *A. gambiae.* Alternatively, they could code for reverse-complement transcripts and represent TE suppression elements. We cannot distinguish between these two possibilities, because the libraries were not directional.

The variant analysis done in the *An. gambiae* midgut transcriptome identified 160,742 variants of which 74.3% are novel with respect to the SNP database. This shows the power of RNA-seq in finding variants and also the high level of polymorphism even within an *An. gambiae* lab colony. A large portion of the variants were found in transcripts that are intergenic with respect to the reference genome, and this class of transcripts have a higher frequency of variants (12.5 variants/transcript) compared with genic transcripts (5.3 variants/transcript), probably due to the higher frequency of non-coding RNAs that are expected to tolerate more variations than protein-coding transcripts.

## Conclusions

The novel midgut transcripts presented here considerably expand the known *An. gambiae* transcriptome. This study sheds light on both coding and polyadenylated non-coding RNAs and their variants, making a significant contribution to the *An. gambiae* genome annotation by doubling the number of known transcripts and tripling the number of variants. Identification and sequence information for many lncRNAs and other novel transcripts opens the possibility to study their transcriptional responses and begin to explore their biologic function in *An. gambiae* using functional assays such as dsRNA gene silencing.

## Methods

### *An. gambiae*and *Plasmodium*parasites

The *An. gambiae* G3 and L3-5 mosquito strains [29] were reared at 27°C, 80% humidity on a 12-h light-to-dark cycle. The *P. falciparum* strains used (3D7 and 7G8) were maintained in O + human erythrocytes using RPMI 1640 medium supplemented with 25 mM Hepes, 50 mg/L hypoxanthine, 25 mM NaHCO_3_, and 10% (vol/vol) heat-inactivated type O + human serum at 37°C and a gas mixture of 5% O_2_, 5% CO_2_, and balance N2 [30, 31]. A green fluorescence protein expressing a *P. berghei* strain (ANKA 2.34) was used and was maintained by serial passages in 3- to 4-week-old female BALB/c mice or as frozen stocks.

### Experimental infection of mosquitoes with *P. falciparum*and *P. berghei*

*An. gambiae* females were infected artificially with *P. falciparum* 3D7 or 7G8 gametocyte cultures. Gametocytogenesis was induced as previously described [32]. Mature gametocyte cultures (stages IV and V) that were 14–16 d were used to feed 4- to 6-day-old female mosquitoes using membrane feeders at 37°C for 30 min. Some midguts were dissected 8 d after feeding to confirm infection, and oocysts were stained with 0.05% (wt/vol) mercurochrome in water and counted by light microscopy. Infection of mosquitoes with *P. berghei* was achieved by feeding on anesthetized infected BALB/c mice. Infectivity of the mice was established by assessing parasitemia and by an exflagellation assay previously described [33]. Mosquito infections were done with mice having parasitemias between 4 and 8% and 2–3 exflagellations/field under 400 × magnifications. Previously infected mosquitoes under permissive (21°C) or non-permissive (28°C) temperatures were infected with *P. berghei* 6 d after the first infection*. P. berghei*-infected mosquitoes were kept at 21°C and 80% humidity, and midguts were collected 24 h after the second infection for RNA extraction. *P. berghei* midgut infection was confirmed 6 d post infection. Mosquito midguts were dissected, fixed for 30 min at room temperature in 4% paraformaldehyde in PBS, mounted in slides with Vectashield mounting media and oocysts were counted under an ultraviolet microscope.

### cDNA library preparation and sequencing

Mosquito midguts were dissected in PBS and stored in RNAlater (Ambion) at −70°C. Total RNA was extracted using TRIZOL (Invitrogen) from at least 30 mosquito midguts for each condition. Quality of total RNA was assessed with an Agilent 2100 Bioanalyser (Agilent). Purification of mRNA and cDNA library preparation was done following the mRNA-Seq sample prep kit (Illumina). Libraries were sequenced after 36 cycles of amplification using an Illumina 1 G genome analyzer according to manufacturer’s instructions. Each sample of amplified material was loaded at a concentration of 4 pM per flow-cell. Raw reads for the four different experimental samples were deposited at the NCBI Sequence Read Archive, (SRA) under accession numbers: SRR1171958, SRR1171976, SRR1172036, and SRR1172037.

### Quality control of Illumina reads

All computational processes were performed in the National Institute of Allergy and Infectious Diseases High-Performance Computing Portal Cluster. Quality trimming of reads was performed with Btrim on four fastq files [12]. A 5-bp window searched for average quality values above 25 as a minimum. The quality filtering continued until a minimum read length of 40 bp was reached; this was used to avoid generating very short reads. Additional file 1: Figure S1A/B shows the quality scores for the Illumina reads after Btrim quality trimming and the distribution of the length of the final reads. This figure was generated using FastQC software (http://www.bioinformatics.babraham.ac.uk/projects/fastqc/). Trimmed fastq files with reads for each condition were then pooled into a single file representative of each mosquito strain, allowing for a greater depth of coverage during the mapping procedure. A heat map of read coverage was generated using SeqMonk (http://www.bioinformatics.babraham.ac.uk/projects/seqmonk/) (Additional file 2: Figure S2A/B) using the base-pair quantitation option. The data zoom was set to 3 on a positive-only linear scale. A boxplot showing the log(FPKM) distribution of G3 and L3-5 samples was generated using the cummeRbund package in R.

### Genome-based analysis of Illumina reads

Mapping of reads to the *An. gambiae* genome (AgamP3.6) was performed using the splice junction mapper TopHat (version 1.3.3) [13]. A reference annotation file from the PEST strain was provided to TopHat during the runs. Because mosquitoes are extremely polymorphic, an initial read mismatch of 3 bp was allowed during the mapping process. For reads spanning splice junctions, the minimum anchor length were set to 10 bp. Transcripts were then designed with Cufflinks version 1.2.1 [15]. Transcript assembly was guided using a reference annotation-based transcript (RABT) assembly [34]. In this approach, the *An. gambiae* PEST genome reference annotation was provided for a more accurate design of novel isoforms of previously known genes. The minimum intron/exon boundary was set to 40 bp [35]. All other parameters were set to default. Assembled transcripts of both the G3 and L3-5 mosquito strains were then independently compared with the annotated reference genome using Cuffcompare, a program packaged with Cufflinks. We chose a normalized read or fragment coverage of FPKM ≥ 1 as a cut-off value for transcripts to be considered reliable for the analysis. The location of mapped reads with respect to previously annotated exons was determined with the BEDTools genome analysis package [36]. BAM files for sequence reads from the *Anopheles gambiae* G3 and L3-5 strains are included as Additional file 9: File S1 and S2, respectively. The genomic location of all TCONS is included as Additional file 9: file S3.

### Transcript design validation

#### Microarray analysis

*An. gambiae* G3 mosquitoes reared under standard laboratory conditions were fed uninfected blood or infected blood with NF54 wild-type *P. falciparum* strain and kept at 27°C. Mosquito midguts were collected in pools of 25 at 12 and 26 h after ingestion of blood, with three biological replicates for each time point. Midguts were placed in 50 μl RNALater (Ambion) in liquid nitrogen and subsequently stored at −70°C until processed. Mosquito midguts were dissected 9–10 d after feeding and stained with 0.1% mercurochrome to confirm infection by determining oocyst numbers. Total RNA was extracted using a modified method involving TRIzol (InVitrogen) and RNeasy mini kit (Qiagen). RNA integrity was determined by an Agilent Bioanalyzer and Agilent 6000 nano assay. A reference design was used to compare all samples to a reference pool of mosquito midguts. Samples were labeled with CY3 and the pooled reference sample labeled with CY5 using the Quick Amp labeling kit (Agilent). Labeled RNA samples were hybridized to a custom designed 4 × 44 K *An. gambiae* microarray (Agilent) consisting of 45,220 probes including 22,287 unannotated transcripts with 60-mer probes designed using e-Array software (Agilent) with the base composition method. Microarrays were scanned with an Agilent G2505C microarray scanner, and image analysis was performed using the Agilent feature extraction method. Entities were filtered separately for each time point based on probe sets as “detected” or “not detected” in at least 1 of 3 biological replicates using Genespring GX 12.5.

#### PCR validation

Primers for PCR validation of selected transcripts in the *An. gambiae* G3 midgut transcriptome were designed using primer3 and custom scripts. PCR was performed with cDNA made with an independent *An. gambiae* G3 midgut sample 24 h after *P. berghei* infection. cDNA was prepared with the QuantiTect reverse transcription kit (Qiagen). For samples that failed the first validation with PCR, a second primer pair was designed and tested. Genomic DNA from G3 mosquitoes was used as a positive PCR control.

#### RNA protein coding potential analysis

Midgut intergenic and nonintergenic transcripts were analyzed with the coding potential assessment tool (CPAT v1.2.1) [17] to determine a coding potential probability score. The *An. gambiae* reference genome (AgamP3.6) was used to calculate the in-frame hexamer frequency table. The default coding potential cutoff of > 0.39 was used to infer high probability of being a coding transcript. Default start and stop codons were used to define ORFs. CPAT predicts coding potential without involving any sequence alignment to databases. Transcripts with coding potential ≤ 0.39 and > 200 bp in length were considered lncRNA [17].

### *De novo*assembly of the Illumina reads and comparison to genome-based analysis

Illumina fastq files for each mosquito strain and condition were pooled into a single all-inclusive library for subsequent analysis. This pooled file went through a genome-based assembly (described above) and a *de novo* assembly using ABySS [18, 19] with variable k values from k = 24 to 96 in steps of 2, or shorter than 96 according to the length of the raw sequences. A limiting qual value, q = 7, was used in all assemblies. The resulting Abyss assemblies were further assembled by a pipeline consisting of blastn and CAP3 [37] iterations as described in [38], consisting of iterations with a decreasing blastn word size inclusion strategy. A master program sequentially sent each transcript to be blasted using an initial word size of 200 (blastn switch -W 200) and a maximum limit of 1,000 matches (−v 1000, using tabular output mode –m 8). Matches were marked as collected as they were retrieved from the blastn program, and these matched sequences were not sent for blastn when their turn arrived, thus avoiding duplicating the BLAST task. The second iteration was done with a word size of 134, the output of which was in turn used for the next round, but now using a word size of 90, then 60, then two more rounds of 48 to produce the final output. The resulting assembled sequences larger than 150 bp were combined into a FASTA file and used as a query against the Cufflinks transcript model database. A BLAST cut-off e-value ≤ 1 × E^−20^ was used as a determinant of a match between programs. The genome-based-transcripts obtained were used as a query and compared with the *de novo* contigs with blastn [34] and vice versa.

### Functional analysis by BLAST

Transcripts with FPKM values ≤ 1 were filtered, and sequences for each individual transcript were extracted from the *An. gambiae* PEST genome. Transcripts were blasted or RPSblasted against several databases and results were mapped to a hyperlinked Excel file, as used before for whole organism's proteomes [39]. A custom automatic classification program screened the BLAST results from databases Swissprot, GO, CDD, Pfam, KOG, SMART, subsets of the non-redundant protein databases, a transposable elements database, and blastn results from an rRNA subset from GenBank to find—based on a vocabulary of approximately 200 words—the best functional class to which a particular transcript could be assigned.

### Variant analysis

BAM files from G3 and L3-5 were preprocessed with Picard and GATK for duplicate marking, sorting, realignment around indels, variant calling, and filtering (http://picard.sourceforge.nethttp://picard.sourceforge.net/) [40]. Variants in positions with a minimum coverage of 20 reads and a strand bias less than −100 in each of the samples were retained for further analysis. Parameters used were based on GATK author’s recommendations (http://www.broadinstitute.org/gatk/guide/topic?name=best-practices). After variants were identified, snpEFF software was used for annotating variants [41]. The integrative genomics viewer was used to visually inspect specific regions of sequence alignments [42].

We defined as “potentially novel” those SNPs not present in the dbSNP version 125.

## Electronic supplementary material

Additional file 1: Figure S1: Quality analysis of Illumina reads for the *An. gambiae* midgut transcriptome. **(A)** Phred quality score for combined reads from *An. gambiae* strains G3 and L3-5 strains after trimming with Btrim. The red horizontal line represents the median quality scores. The yellow boxes display the interquartile range (25th – 75th percentile). Whiskers display the largest and smallest values. The blue line represents the mean quality score. The background green area represents very good quality scores. The background orange area represents reasonable quality scores. The background red area represents poor quality scores. The quality threshold was set at 25 or more. **(B)** Number of Illumina reads by length for *An. gambiae* after quality trimming. **Figure S2.** Frequency of *An. gambiae* midgut transcripts by FPKM. Low coverage transcripts with FPKM ≤1 in either G3 or L35 *An. gambiae* strains were removed from the downstream analysis of the midgut transcriptome. **Figure S3.** FPKM distribution of *Anopheles gambiae* G3 and L35 srain transcripts. Log (FPKM) of all transcripts in G3 (blue) and L35 (brown) mosquitoes. Black horizontal line represents median values. The hinges correspond to the first and third quartiles (the 25th and 75th percentiles). The upper and lower whiskers display the largest and smallest values that are not outliers. Black dots represent outliers. **Figure S4.** Genome mapping of the sequence reads of three novel intergenic transcripts (NITs) that were experimentally validated. Primers sequences to validate transcript expression are shown in Table S3. **Figure S5.** Graphic respresentation of the genomic location of three new exons in a predicted cDNA (TCONS_00023667) for the *Anopheles gambiae* cyclin A gene (AGAP012413). Primers were designed between exons and the PCR products were sequenced to confirm the predicted splice junctions. Primer sequences are shown in Additional file 3: Table S4. (DOCX 2 MB)

Additional file 2: Table S1: Sequence of the *Anopheles gambiae* midgut transcripts identified by genome-based analysis (FastA format). Accessible in http://exon.niaid.nih.gov/Agam_MGT_RNAseq/. **Table S2**. Functional annotation of *Anopheles gambiae* midgut transcripts identified by genome-based analysis. Accessible in http://exon.niaid.nih.gov/Agam_MGT_RNAseq/. (ZIP 74 MB)

Additional file 3: Table S3: Validation of transcript expression by qRT-PCR was done in an independent *A. gambiae* midgut sample. The primers used, amplicon size, Cufflinks class code, FPKM and detection results are indicated. (PDF 65 KB)

Additional file 4: Table S4: Validations of novel intron-exon splice variants in the *Anopheles gambiae* midgut transcriptome. Validation of novel intron-exon splice variants by qRT-PCR was done in an independent *A. gambiae* midgut sample. The primers used, amplicon size, Cufflinks class code, FPKM and detection results are indicated. (PDF 52 KB)

Additional file 5: Table S5: Microarray validation of novel transcript expression from the *Anopheles gambiae* midgut. List of microarray probes used for the validation of novel intergenic *An. gambiae* midgut transcripts. The probes with a positive signal are indicated as “Detected”. (XLSX 143 KB)

Additional file 6: Table S6: Functional classification of *An. gambiae* midgut transcripts from a genome-based analysis. Previously annotated Cufflinks transcripts correspond to a complete match to the reference annotation. Potentially novel transcripts encompass every other Cufflinks transcript class (see Table 1). “RNA proc.,Transc & Transl”: RNA Processing, Transcription, Translation; “Cytosk/Stor/Secr/ExMtrx”: Cytoskeletal, Secretion, Extracellular Matrix; “Post transl mod/Prot mach”: Post-translational modification and proteasome machinery; “Peptidases & Prot. inhibitors”: Protease and protease inhibitors. (PDF 362 KB)

Additional file 7: Table S7: Sequence of the *Anopheles gambiae* midgut transcripts identified by de novo analysis (FastA format). Accessible in http://exon.niaid.nih.gov/Agam_MGT_RNAseq/. **Table S8.** Functional annotation of *Anopheles gambiae* midgut transcripts identified by *de novo* analysis. Accessible in http://exon.niaid.nih.gov/Agam_MGT_RNAseq/. **Table S9.** Variants in the *Anopheles gambiae* midgut transcripts relative to the reference genome (PEST strain). Accessible in http://exon.niaid.nih.gov/Agam_MGT_RNAseq/. (ZIP 211 MB)

Additional file 8: Table S10: Variants per expressed gene in *Anopheles gambiae* midgut transcripts. (XLSX 324 KB)

Additional file 9: File S1: BAM files for sequence reads from Anopheles gambiae G3 strain. Accessible in http://exon.niaid.nih.gov/Agam_MGT_RNAseq/. **File S2.** BAM files for sequence reads from Anopheles gambiae L3-5 strain. Accessible in http://exon.niaid.nih.gov/Agam_MGT_RNAseq/. **File S3.** Genomic location of transcript models (TCONS). Accessible in http://exon.niaid.nih.gov/Agam_MGT_RNAseq/. (ZIP 2 MB)

## Abbreviations

ABySS: Assembly by short sequences
CPAT: Coding potential assessment tool
EST: Expressed sequence tag
FPKM: Fragment per kilobase of exon per million fragments mapped
lncRNA: Long non-coding RNA
NIT: Novel intergenic transcript
ORF: Open reading frame
R: Resistant
PEST: Pink-eyed laboratory strain of *An. gambiae*
RABT: Reference annotation-based transcript
RT-PCR: Reverse transcript-polymerase chain reaction
S: Susceptible
snpEFF: A program for annotating and predicting the effects of single-nucleotide polymorphisms.
