# Supplementary material for: In depth annotation of the Anopheles gambiae mosquito midgut transcriptome
Source: BMC Genomics. 2014 Jul 29;15(1):636. doi: 10.1186/1471-2164-15-636 (PMC4131051; doi:10.1186/1471-2164-15-636)
Supplement: Supplementary file 1 — Additional file 1: Figure S1: Quality analysis of Illumina reads for the An. gambiae midgut transcriptome. (A) Phred quality score for combined reads from An. gambiae strains G3 and L3-5 strains after trimming with Btrim. The red horizontal line represents the median quality scores. The yellow boxes display the interquartile range (25th – 75th percentile). Whiskers display the largest and smallest values. The blue line represents the mean quality score. The background green area represents very good quality scores. The background orange area represents reasonable quality scores. The background red area represents poor quality scores. The quality threshold was set at 25 or more. (B) Number of Illumina reads by length for An. gambiae after quality trimming. Figure S2. Frequency of An. gambiae midgut transcripts by FPKM. Low coverage transcripts with FPKM ≤1 in either G3 or L35 An. gambiae strains were removed from the downstream analysis of the midgut transcriptome. Figure S3. FPKM distribution of Anopheles gambiae G3 and L35 srain transcripts. Log (FPKM) of all transcripts in G3 (blue) and L35 (brown) mosquitoes. Black horizontal line represents median values. The hinges correspond to the first and third quartiles (the 25th and 75th percentiles). The upper and lower whiskers display the largest and smallest values that are not outliers. Black dots represent outliers. Figure S4. Genome mapping of the sequence reads of three novel intergenic transcripts (NITs) that were experimentally validated. Primers sequences to validate transcript expression are shown in Table S3. Figure S5. Graphic respresentation of the genomic location of three new exons in a predicted cDNA (TCONS_00023667) for the Anopheles gambiae cyclin A gene (AGAP012413). Primers were designed between exons and the PCR products were sequenced to confirm the predicted splice junctions. Primer sequences are shown in Additional file 3: Table S4. (DOCX 2 MB) [file 12864_2014_6335_MOESM1_ESM.docx]

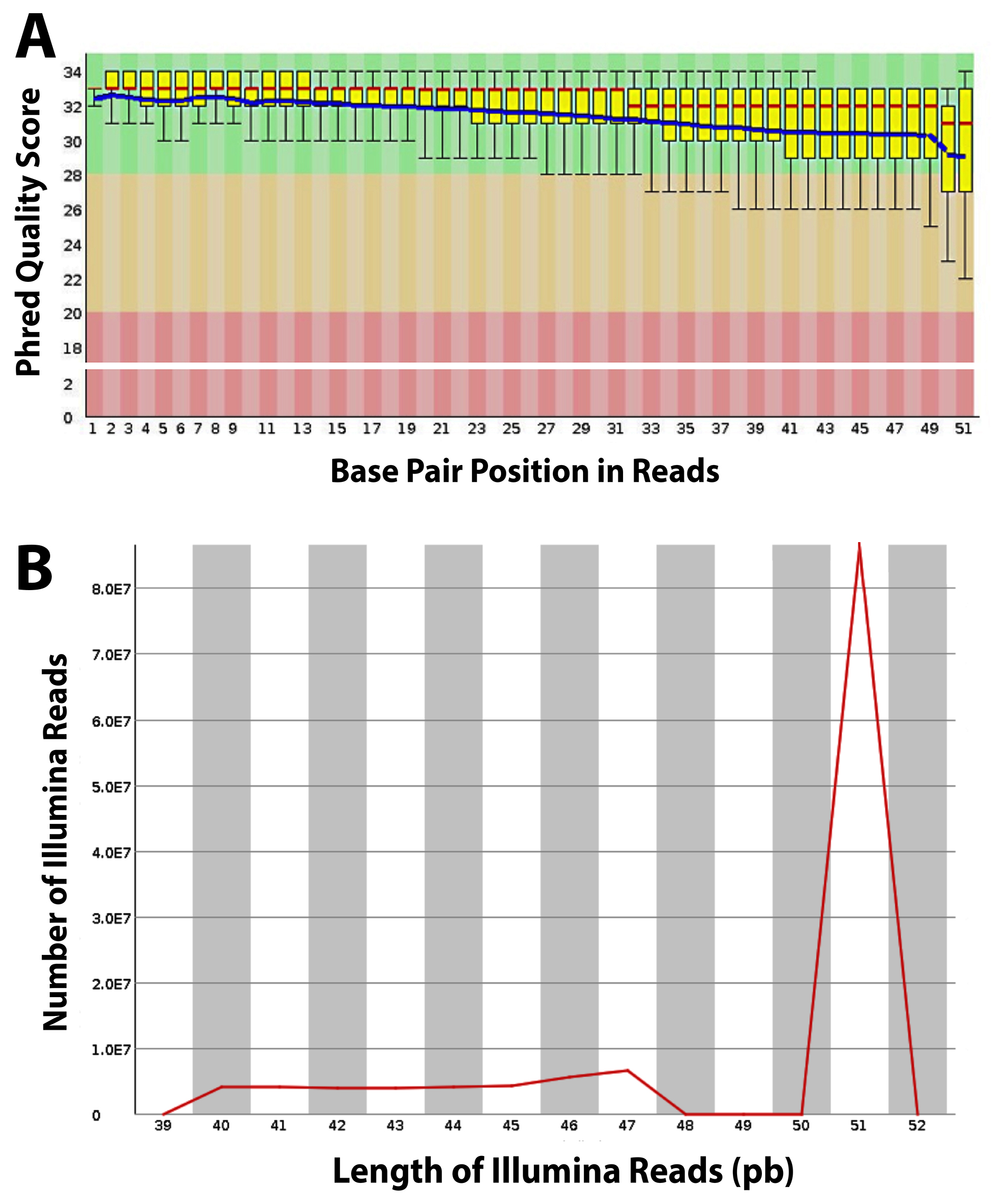


**Figure S1.** Quality analysis of Illumina reads for the *An. gambiae* midgut transcriptome. (A) Phred quality score for combined reads from *An. gambiae* strains G3 and L3-5 strains after trimming with Btrim. The red horizontal line represents the median quality scores. The yellow boxes display the interquartile range (25^th^ – 75^th^ percentile). Whiskers display the largest and smallest values. The blue line represents the mean quality score. The background green area represents very good quality scores. The background orange area represents reasonable quality scores. The background red area represents poor quality scores. The quality threshold was set at 25 or more. (B) Number of Illumina reads by length for *An. gambiae* after quality trimming.


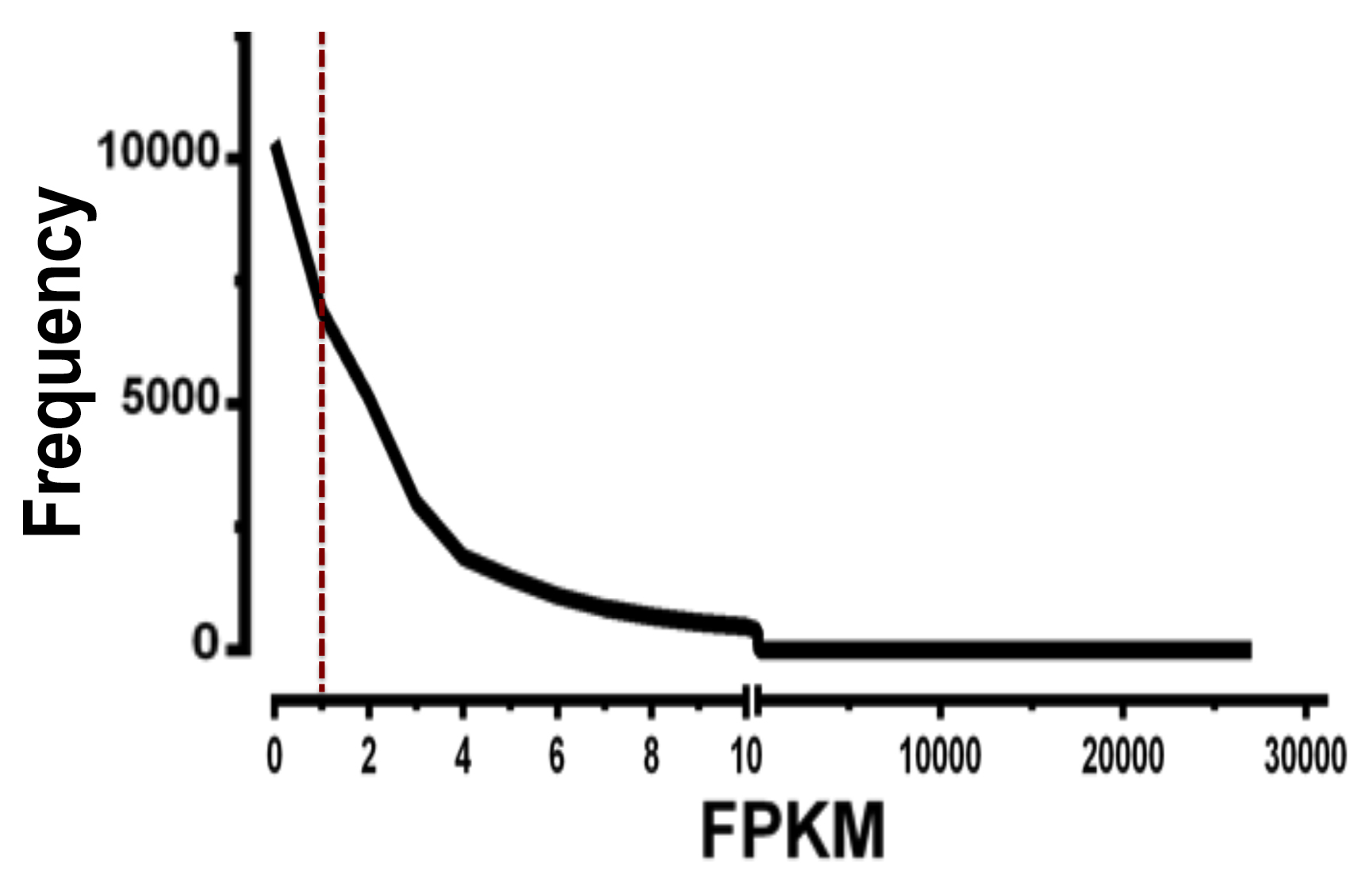


**Figure S2.** Frequency of *An. gambiae* midgut transcripts by FPKM. Low coverage transcripts with FPKM ≤1 in either G3 or L35 *An. gambiae* strains were removed from the downstream analysis of the midgut transcriptome.


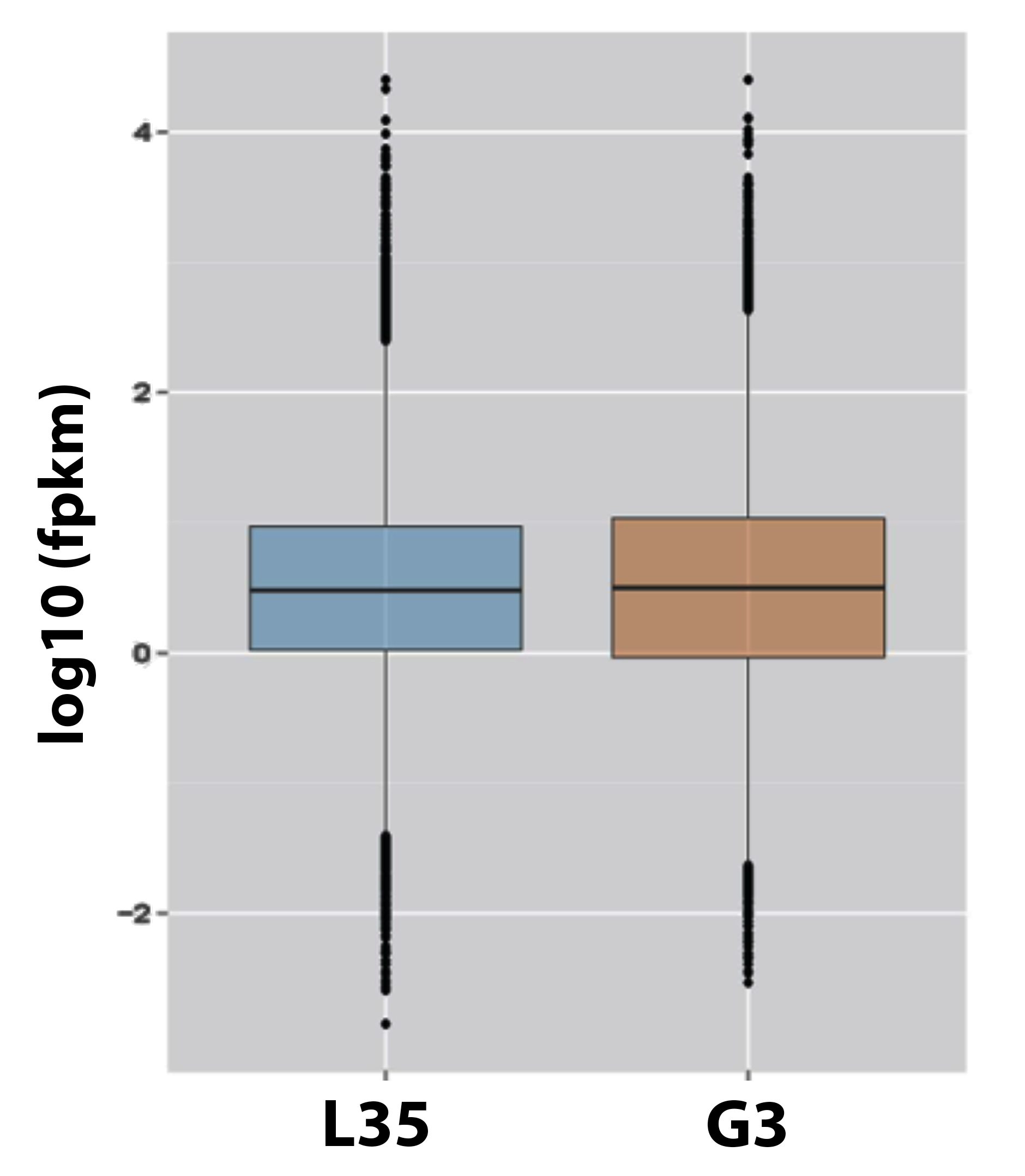


**Figure S3.** FPKM distribution of *Anopheles gambiae* G3 and L35 srain transcripts. Log(FPKM) of all transcripts in G3 (blue) and L35 (brown) mosquitoes. Black horizontal line represents median values. The hinges correspond to the first and third quartiles (the 25^th^ and 75^th^ percentiles). The upper and lower whiskers display the largest and smallest values that are not outliers. Black dots represent outliers.


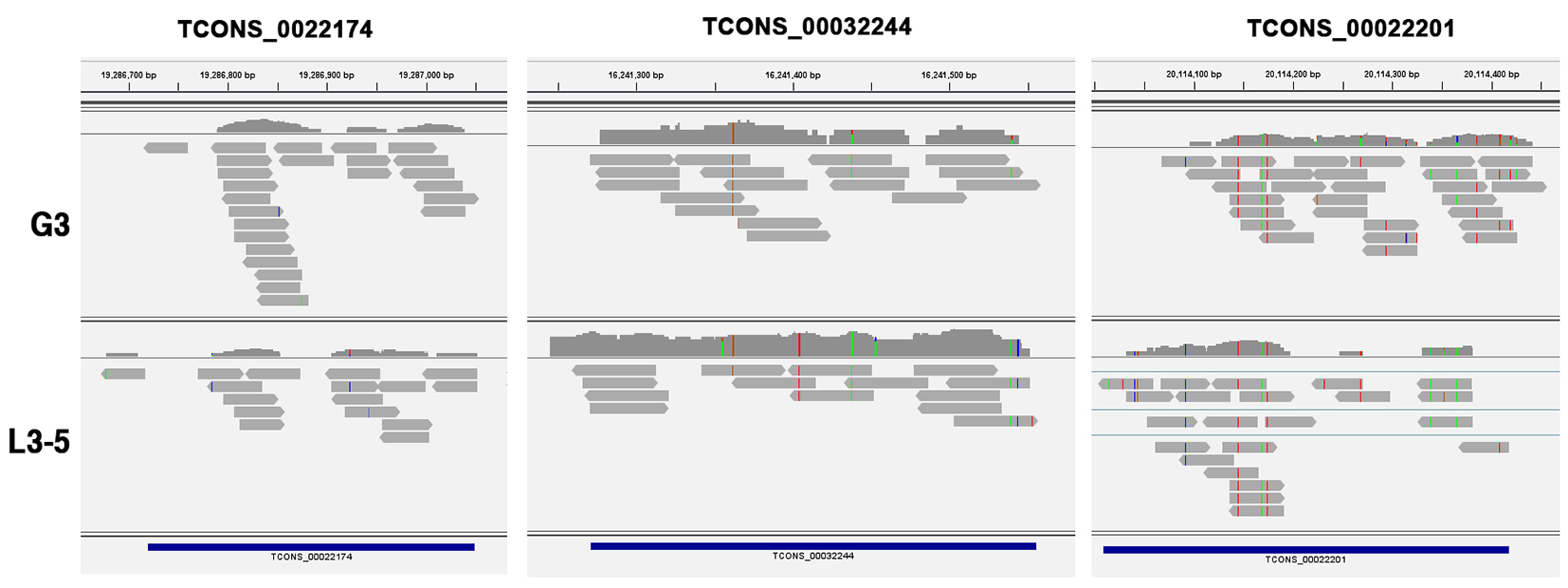


Figure S4. Genome mapping of the sequence reads of three novel intergenic transcripts (NITs) that were experimentally validated. Primers sequences to validate transcript expression are shown in Table S3.


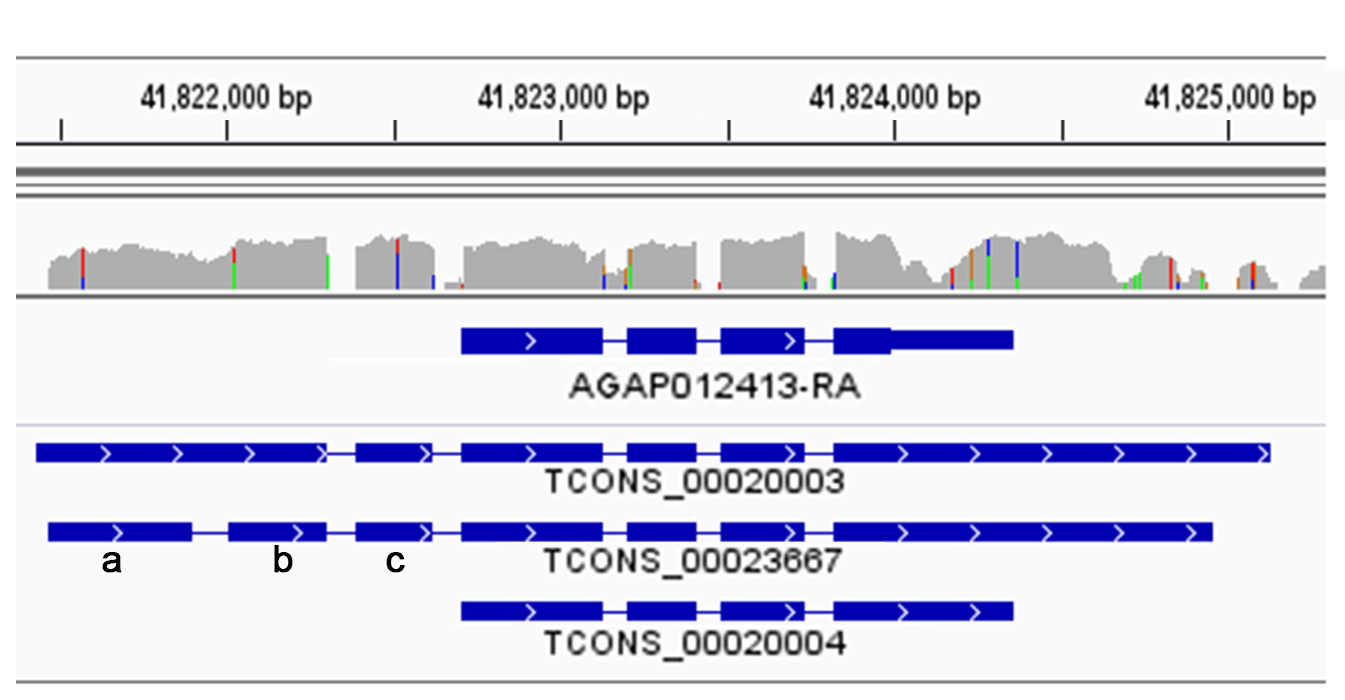


Figure S5. Graphic respresentation of the genomic location of three new exons in a predicted cDNA (TCONS_00023667) for the *Anopheles gambiae* cyclin A gene (AGAP012413). Primers were designed between exons and the PCR products were sequenced to confirm the predicted splice junctions. Primer sequences are shown in Table S4.
