# Supplementary material for: In depth annotation of the Anopheles gambiae mosquito midgut transcriptome
Source: BMC Genomics. 2014 Jul 29;15(1):636. doi: 10.1186/1471-2164-15-636 (PMC4131051; doi:10.1186/1471-2164-15-636)
Supplement: Supplementary file 3 — Additional file 3: Table S3: Validation of transcript expression by qRT-PCR was done in an independent A. gambiae midgut sample. The primers used, amplicon size, Cufflinks class code, FPKM and detection results are indicated. (PDF 65 KB) [file 12864_2014_6335_MOESM3_ESM.pdf]

Table S3. Validation of expression of *Anopheles gambiae* midgut novel transcripts by qRT-PCR.

Validation of transcript expression by qRT-PCR was done in an independent *A. gambiae* midut sample. The primers used, amplicon size Cufflinks class code, FPKM and detection results are indicated.

Transcript ID	Primer F	Primer R	Amplicon Size	Class code	FPKM	Detected?
TCONS_00006299	CAATTTGCACACACAATAAAC	TAAGCAGAACAAAGTGACATTAG	150	o	0.306869	Yes
TCONS_00003756	ATAAACACATACGAACAGAGAGAC	TTGTGAGCTCTTTTGTTGAC	150	u	0.393754	Yes
TCONS_00011544	TCTCTCTCTCTCTCTCTCTCTTTAC	ACTACCCTGTAGAAAGTCAACTAA	150	o	0.461712	Yes
TCONS_00024341	ATTTGGATGGTAATACTGCATAC	TGTTATACATGTTTCATTTCGTG	150	u	0.463521	Yes
TCONS_00005711	GAAAATGAAGAAGGTGAAGAAC	GACAATCGGTTCTCTCTCAG	150	e	0.506611	Yes
TCONS_00009632	CAGCGTCAATTACGATACG	GATAGCTCCGATAATATTTCCTC	150	u	0.530405	Yes
TCONS_00019129	TAAAAACGGAAGTGAATGAG	GAACGATAAACTACGATACGAG	150	u	0.55613	Yes
TCONS_00000582	ACTACAAAATTGCATCTCTATCTG	TTCTTTGTATTACGTCTACTTTACG	150	e	0.559452	Yes
TCONS_00011333	GGTGTGTATGTGTGAGTGTATTTG	GTATTTAAGCCCTTTGTTAACTTG	150	u	0.592527	No
TCONS_00005591	TATATACTGTGCGATGATTATATGG	ACAAAGAATATACTCCTTTAGATG	150	p	0.808216	No
TCONS_00005686	TTCCATTTACTAACAACTGTAACTC	TATATGAAGTCCAACTCCAGTG	150	p	0.86968	Yes
TCONS_00024038	CATATACAGTCTCAGATGATAAAAC	AGTGTCACCTAGAGACGAAG	150	u	0.884429	No
TCONS_00017003	AAAATCTTAATCGAAATCGTCTC	GATTCTGTACCTGATTTTGACTC	150	p	0.936899	Yes
TCONS_00021911	GTACTCTGTACTGTACTCGACTG	AGTTCATCCTAACAACATACAC	149	u	0.989295	Yes
TCONS_00024695	TTAATAAAACACACACACACAC	TATACTTGGGTAATGCACAG	151	u	0.992318	Yes
TCONS_00007720	AATCTAGAGAGTACAATCGAAAC	CGTAGTCGAGAAATAGATTTAG	148	u	1.019166	Yes
TCONS_00024309	ACTCGTATGACTTAACAACATGAAC	AAATTGTCTCAATATGTCTGGTTAC	150	u	1.05593	Yes
TCONS_00024266	CATACCTACGAAAAACCAGTG	GATGTCAAAATGTTTTACTTCAC	150	p	1.146248	Yes
TCONS_00012845	TAATTACGTTCATAAGCTAACAC	CGGTGGTATAGTGATGATG	150	u	1.180639	Yes
TCONS_00007648	GTGTAGAATGTGTTGTTTATTG	CTTAGGGTAACTAACGCTATC	150	u	1.197861	Yes
TCONS_00005769	TTTTAGGGTTTTTGTTGTTGTTG	TATGTTCCGTTGATTTATAATCCC	150	i	1.333727	Yes
TCONS_00024315	CTACGGTTCATCAAGTGC	ATAACACTCCACCACTATCC	150	u	1.384962	Yes
TCONS_00009216	ACTAGCAAACACAATCGTC	GATTTGTTTCTTTTTGCTG	150	u	1.407028	Yes
TCONS_00021375	AACACGTAGAAATAAACAAGG	GCTGTAACTGAAAGTTGACTC	150	u	1.496962	Yes
TCONS_00023257	GACATCTTGAATGTGATTATGTG	CTCTCTCTCTCTTTCTGTCTCTC	150	u	1.521614	No
TCONS_00034103	TTTCTACGGTAGCTTTACTTC	ACATGAACATGATTGTATTCC	150	u	1.602879	Yes
TCONS_00017437	GGAATAGCGTTGATTTAGC	CACTGATATGTAGAGTGTTGTAGA	149	u	1.633662	Yes
TCONS_00013184	AACTCACACATACACACTCAAAG	AAACTGATAAGAGACTGCTACTAC	600	j	1.645551	Yes
TCONS_00005060	ATGAGTTAAACATATGGAAAGAC	GTTATCGTGTACATTTACTTTTC	149	u	1.648681	No
TCONS_00005000	TGAGCTTATACTAGTTCTTTTGG	GGTTAGTTTAATACATACCAACATC	150	u	1.652122	Yes
TCONS_00005001	TTCTACTGGTTCTATTATTACCG	ATCTTGCTTTCTTGATCTTTAC	150	u	1.702571	Yes
TCONS_00018857	GTATCGCATTACTCGAAAATAATAG	TCAGCTGTCTTATTCTTCTTCTTC	600	j	1.715457	Yes
TCONS_00009945	CTGAAGCATCTGATGAACTACTAC	GACGTACTCTTCGTTGAGG	600	j	1.813174	Yes
TCONS_00028506	TAACAAAGCACTACCTCAAGC	AGGTGACTTCACACTTGTCC	600	=	1.836078	Yes
TCONS_00010621	CTATTGCAAATCGTGTAGTATATG	GTGTTCCACTAGTCTTACAAAATC	599	i	1.870046	Yes
TCONS_00036217	GATGAAGAGGAAGATGACGAG	GAACAGGACAGGGACCAG	600	=	1.968085	Yes
TCONS_00030238	GTTTCAGATTTCTTTTCTCATTC	GTCTGAAATTAACGAGCTGTAG	600	=	2.033674	Yes
TCONS_00014056	GAGATAATGGACACATATCAGAAG	ACTTTCACCATCTGAAGCTATAC	601	=	2.064097	Yes
TCONS_00022107	GTATCGCTTTATATATTGTTAGCAC	TATTTTATAACGACGATGTGTTG	599	p	2.175829	No
TCONS_00016970	TGTAGTCTAAATCTCTTTCCCTTAG	GTCTGCTACATAAAACAAAAACTG	600	j	2.221265	Yes
TCONS_00001231	TCGTTTTGATAAACTCGATAAC	TATCTGTTTAAGAACGTGTTCAG	601	=	2.320936	Yes
TCONS_00007415	GAGAAGTACATATATTGCATACC	CATTGTTTACCATCGTACC	150	u	2.328564	Yes
TCONS_00016414	CTAAAAATGCTTCCCAATG	ATACATTGGTAGCGAAATGAC	600	=	2.431768	No
TCONS_00032866	GGCAGATATAAGATGATAACG	GCCCTGTACATTTCAAAC	149	u	2.465282	Yes
TCONS_00022447	TCTAGGAGGAGATGTACCAG	CCAGTAATGGTCTAATAAATTG	149	u	2.495375	Yes
TCONS_00016620	ATTGCGATAAAAGGCAAC	ATCTTAATGATGTCCTTGAACTC	604	j	2.552828	Yes
TCONS_00005525	ATGCTATCGATTTATGTTAGTC	GTACGATGTACAGCATAAGAG	151	u	2.55412	No
TCONS_00005695	GTTAGATTTGGTAATGTTGACTC	GAGAGAGAGAGAGAGAGAGAGA	150	u	2.641962	No
TCONS_00025373	ATTCAGTGTGACATACATATCC	GTTCGTTTCATTTTGTCG	154	u	2.673924	Yes
TCONS_00016462	CGACGGCTTTTGATTTCGTA	GGAAAATCAGGTGTGCCAGT	425	i	2.733234	Yes
TCONS_00022002	GTAGGAAAAAGGGGGTGAGC	CCGCATTCAATTTTGGAGTC	226	u	3.052776	Yes
TCONS_00006269	GCCTTGCTCGAGTATGTACGA	GTCCAATACCGCCGAGAGT	304	u	3.310948	Yes
TCONS_00017751	AAGTCGACGGATTACCACGA	CCGTTCATTTTCGATCAACC	203	u	3.495017	Yes
TCONS_00032244	GATTGCAGCAACAGAGATAGC	AGAACGCAACACGCATACAG	205	u	3.692191	Yes
TCONS_00021987	CCGCTATCGAATCTTTGGAG	GCTACGCTGACGAAAAAGTACA	226	u	3.733785	Yes
TCONS_00027665	GCGGTTATTCTTTGTCGTTG	GCAGAATGGTTGGGATATTG	203	u	4.170971	Yes

* Cufflinks class codes:

= Complete match of intron chain.
c Contained.
j Potentially novel isoform (fragment): at least one splice junction is shared with a reference transcript.
e Single exon transfrag overlapping a reference exon and at least 10 bp of a reference intron, indicating a possible pre-mRNA fragment.
i A transfrag falling entirely within a reference intron.
o Generic exonic overlap with a reference transcript.
p Possible polymerase run-on fragment (within 2Kbases of a reference transcript).
r Repeat. Currently determined by looking at the soft-masked reference sequence and applied to transcripts where at least 50% of the bases are lower case.
u Unknown, intergenic transcript.
x Exonic overlap with reference on the opposite strand.
s An intron of the transfrag overlaps a reference intron on the opposite strand (likely due to read mapping errors).
. tracking file only, indicates multiple classifications.
