# Supplementary material for: In depth annotation of the Anopheles gambiae mosquito midgut transcriptome
Source: BMC Genomics. 2014 Jul 29;15(1):636. doi: 10.1186/1471-2164-15-636 (PMC4131051; doi:10.1186/1471-2164-15-636)
Supplement: Supplementary file 4 — Additional file 4: Table S4: Validations of novel intron-exon splice variants in the Anopheles gambiae midgut transcriptome. Validation of novel intron-exon splice variants by qRT-PCR was done in an independent A. gambiae midgut sample. The primers used, amplicon size, Cufflinks class code, FPKM and detection results are indicated. (PDF 52 KB) [file 12864_2014_6335_MOESM4_ESM.pdf]

Table S4. Validation of novel intron-exon splice variants in the *Anopheles gambiae* midgut transcriptome.
Validation of novel intron-exon splice variants by qRT-PCR was done in an independent *A. gambiae* midgut sample.
The primers used, amplicon size, Cufflinks class code, FPKM and detection results are indicated.

Transcript ID	Primer F	Primer R	Amplicon Size	Class code	FPKM	Detected?
TCONS_00027482	CTGGTTATAAGCATGGCCAAA	CACCTTGTCTTGCTAAATTGC	150	j	0.0678	No
TCONS_00027565	CGAGGAAATCCTACCGTTGA	TTGTTGCACACCCTTTCCTA	150	j	0.0923	No
TCONS_00027489	CCACCGCACTATCTCTTCTTG	ATCAGCAGCAGCAAAGGAAG	150	j	0.345	No
TCONS_00005218	GACCGGATGCTGAACCACTA	AGCAGCAGCAACAACAACAG	150	j	0.417051	Yes
TCONS_00011735	GGAACTGGTGGACGGTACTC	AAGCGAAACACCTGTTGCAT	150	j	0.429486	Yes
TCONS_00004941	CCAAAGGATGCGAATAGACC	GTTTGTTTCTATTCATAACTGCACTG	150	j	0.437805	Yes
TCONS_00001332	CTTCGTGAACAGCATGTACTCC	GGTGCCAATCACGTCCAG	150	j	0.444943	Yes
TCONS_00013786	GGATGCAAATCGAGTACAGAAA	GCTTCCGGTCAATGTCTAGC	150	j	0.449991	Yes
TCONS_00011161	AGACAGGCTGTTCGACGTTT	CCTTGAAGATAAACAATCAACAAA	150	j	0.474432	Yes
TCONS_00020202	TTTCGGAATCTTTCGCATTT	TGGAAACCAATGCTGATTTG	150	j	0.594295	Yes
TCONS_00029731	AGTGATCGTAATCGAAGCACA	GCACGCTTTTCTTTCCTTTTT	150	j	0.650438	Yes
TCONS_00021567	ATGGCTATCAGCGAGATGG	GTGCAGTGAGAATGGGGAAT	150	j	0.724242	Yes
TCONS_00026189	AGCAACTCGTGCTGGTAAGC	GCTGAAGATAAGCGAGCTCAA	150	j	0.925491	Yes
TCONS_00031675	CTTGTTCGGTTTGTCGATGA	AACCACGGTGGCACCTATT	150	j	1.050692	Yes
TCONS_00027557	TCTCCGGCACAAGATGATG	AGCACATCACAAAACCCACA	150	j	1.254	No
TCONS_00023693	CCCATACAGCCGAGAAACAT	TCACGCAAATGATGACGAAG	150	j	1.475705	Yes
TCONS_00027440	CCGATGAAGTTGTCCAGGAT	GATACTGGCGCTCACCAAAC	150	j	2.013	No
TCONS_00028689	GACGTCGTTGCGGAATTACT	CATCAACAAAGCAGCAGCAC	150	j	2.733843	Yes
TCONS_00031369	CTACCGGGCCATTATTCGT	GATACTTCCTCCCGCTGCT	150	j	3.817812	Yes
TCONS_00023667a	TGCAAGATAGACGCAATTGAA	CGCCATTTTTAACACTATTCTGA	150	j	3.97981	Yes
TCONS_00023667b	TCAGCGTTCTCAGCAATGTC	TCTGCTTAGCTTCCACAGCA	150	j	3.97981	Yes
TCONS_00023667c	ACCATTCAAAGGGTTCGAGA	CCCACTGACATGGGAGTTTC	150	j	3.97981	Yes
TCONS_00031187	CAAACAATAGGGGGAAAGAGC	GTGTAAGCGCCATCAAAGC	150	j	4.592884	Yes
TCONS_00015197	GCAATTCTGGTTGCCTTTTT	CAAAGTTCCTTGGATTGAAGTG	150	j	4.94972	Yes
TCONS_00001593	GCGCTTCATCCATTTGAAAG	GCTAAATGTAGCGGCCAGAA	150	j	5.13354	Yes
TCONS_00038198	CTGCTCCAGCTCCTTCTGC	AGCTCCGAGGAGGATGAAG	150	j	6.507286	Yes
TCONS_00026773	GTCCGCGTGAACATTTGC	GTACTTGACCGGACGCATCT	150	j	6.714745	Yes
TCONS_00028562	CTGCTGCTGCTGCTGCTT	CCACTTCCCTGTTAGTGCGTA	150	j	6.800724	Yes
TCONS_00001007	TCGTGGTGTACTGACGCACT	GGAGCAGGGGAAATTTTGTA	150	j	7.56145	Yes
TCONS_00018911	GCTTACGCTACAGCATCGTG	ATCGCTTCCGAGTAGAGATGC	150	j	30.586961	Yes
TCONS_00018644	AAACAGCCAACATGCGAAAC	CCAGGTTGCGTTCGTACAG	150	j	69.855885	Yes

* Cufflinks class codes:

j Potentially novel isoform (fragment): at least one splice junction is shared with a reference transcript.
