# Supplementary material for: In depth annotation of the Anopheles gambiae mosquito midgut transcriptome
Source: BMC Genomics. 2014 Jul 29;15(1):636. doi: 10.1186/1471-2164-15-636 (PMC4131051; doi:10.1186/1471-2164-15-636)
Supplement: Supplementary file 6 — Additional file 6: Table S6: Functional classification of An. gambiae midgut transcripts from a genome-based analysis. Previously annotated Cufflinks transcripts correspond to a complete match to the reference annotation. Potentially novel transcripts encompass every other Cufflinks transcript class (see Table 1). “RNA proc.,Transc & Transl”: RNA Processing, Transcription, Translation; “Cytosk/Stor/Secr/ExMtrx”: Cytoskeletal, Secretion, Extracellular Matrix; “Post transl mod/Prot mach”: Post-translational modification and proteasome machinery; “Peptidases & Prot. inhibitors”: Protease and protease inhibitors. (PDF 362 KB) [file 12864_2014_6335_MOESM6_ESM.pdf]

Table S6

Functional Class	Transcripts					
	Total	%	Prev. Annot.	%	Potential Novel	%
Unknown	12981	56.71	1079	19.68	11902	68.38
RNA Proc., Transc & Transl	2054	8.97	983	17.93	1071	6.15
Signal Transduction	1705	7.45	581	10.60	1124	6.46
Metabolism	1590	6.95	898	16.38	692	3.98
Cytosk, Stor, Secr, Extr Mtrx	1124	4.91	413	7.53	711	4.08
Transporters and Channels	596	2.60	248	4.52	348	2.00
Post Transl Mod & Prot Mach	574	2.51	313	5.71	261	1.50
Nuclear Export and Regulation	559	2.44	255	4.65	304	1.75
Protein Export Machinery	541	2.36	264	4.81	277	1.59
Peptidases & Prot. Inhibitors	275	1.20	137	2.50	138	0.79
Transposable elements	243	1.06	14	0.26	229	1.32
Apoptosis	222	0.97	79	1.44	143	0.82
Detoxification	216	0.94	127	2.32	89	0.51
Immunity	204	0.89	92	1.68	112	0.64
Viral product	5	0.02	0	0.00	5	0.03
Total	22889	100	5483	100	17406	100

Table. S6. Functional classification of *An. gambiae* midgut transcripts from a genome-based analysis. Previously annotated Cufflinks transcripts correspond to a complete match to the reference annotation. Potentially novel transcripts encompass every other Cufflinks transcript class (see Table 1). “RNA proc.,Transc & Transl”: RNA Processing, Transcription, Translation; “Cytosk/Stor/Secr/ExMtrx”: Cytoskeletal, Secretion, Extracellular Matrix; “Post transl mod /Prot mach”: Post-translational modification and proteasome machinery; “Peptidases & Prot. inhibitors”: Protease and protease inhibitors.
